# Characterization of In-Body to On-Body Wireless Radio Frequency Link for Upper Limb Prostheses

**DOI:** 10.1371/journal.pone.0164987

**Published:** 2016-10-20

**Authors:** Antonietta Stango, Kamya Yekeh Yazdandoost, Francesco Negro, Dario Farina

**Affiliations:** 1 Institute of Neurorehabilitation Systems, Bernstein Focus Neurotechnology Goettingen, Bernstein Center for Computational Neuroscience, University Medical Center Goettingen, Georg-August University, 37075, Goettingen, Germany; 2 Centre for Wireless Communications, Department of Communications Engineering, University of Oulu, P.O. Box 4500, 90014, Oulu, Finland; 3 Department of Clinical and Experimental Sciences, University of Brescia, 25123, Brescia, Italy; Shanghai Jiao Tong University, CHINA

## Abstract

Wireless implanted devices can be used to interface patients with disabilities with the aim of restoring impaired motor functions. Implanted devices that record and transmit electromyographic (EMG) signals have been applied for the control of active prostheses. This simulation study investigates the propagation losses and the absorption rate of a wireless radio frequency link for in-to-on body communication in the medical implant communication service (MICS) frequency band to control myoelectric upper limb prostheses. The implanted antenna is selected and a suitable external antenna is designed. The characterization of both antennas is done by numerical simulations. A heterogeneous 3D body model and a 3D electromagnetic solver have been used to model the path loss and to characterize the specific absorption rate (SAR). The path loss parameters were extracted and the SAR was characterized, verifying the compliance with the guideline limits. The path loss model has been also used for a preliminary link budget analysis to determine the feasibility of such system compliant with the IEEE 802.15.6 standard. The resulting link margin of 11 dB confirms the feasibility of the system proposed.

## Introduction

In the last decade, there has been an increasing need of acquiring biometric signals for monitoring vital signs and supporting chronically ill patients. In particular, implanted devices can be used to monitor and diagnose cardiac pathologies, cancer, asthma and neurological disorders [1]. More recent applications of wireless implanted systems focus also on helping people with physical disabilities [2], e.g. cochlear and retina implants, or active limb prostheses [3]. This study investigates the possibility to use implanted devices able to record and transmit electromyogram (EMG) signals in order to allow amputees to control active myoelectric prostheses.

The active prostheses currently available in the market have multiple Degrees of Freedom (DoFs) and are usually driven by surface EMG signals. Surface EMG can be easily detected non-invasively with a relatively large pick up area [4]. However, due to the donning/doffing of the prosthesis, the surface EMG electrodes in the socket may change position with respect to the underlying muscles compromising the reliability of the system. Moreover, surface EMG can only be recorded from superficial muscles and they can be prone to changes in skin impedance and breakage of the wires [5]. Intramuscular electrodes may limit these problems when coupled with wireless transmission. Therefore, recently, the use of implanted devices which can wirelessly transmit the EMG signals to a controller embedded in the socket of the prosthesis has been proposed [5, 6]. Implanted EMG sensors can provide information from deep muscles that are not easily accessible with standard surface EMG electrodes, and therefore it can help to improve the control of the prosthesis.

In [6] the authors propose a system with two implanted electrodes, which use a transmission protocol tailored for recording physiological signals. The RF link is based on IEEE 802.15.4 [7] in the 2.4 GHz frequency band, while the power transfer is done by an inductive link. In the most detailed study in this field [5], the authors present a multichannel system that can receive and process signals from EMG implanted sensors. The system also comprises a large external power coil, placed around the limb, a receiving antenna, and a telemetry device which passes the data to the prosthesis controller. This system has been recently implanted in a patient [8] and improved [9], but it has some limitations: the large inductive power field lowers its overall efficiency and there is no flexibility in the location of the implants since they must be within the inductive area and parallel to the external coil. Furthermore the presence of a circular coil limits the use of such system to amputees with a round stump [10].

The radio communication between implanted biosensor and external biomedical systems has specific issues due to the fact that the human body is a heterogeneous propagation environment. The human body is characterized by multiple layers of tissues with different thicknesses and dielectric properties. Furthermore, every application demands devices with appropriate shapes and requirements, depending on the place where they have to be implanted and on the tissues involved in the transmission [11]. The antenna shape and dimensions depend on the application and on the band of operation [11]. The band assigned by the Federal Communication Commission (FCC) and accepted in most Countries for implanted communication is 402–405 MHz [12, 13]. This band is called medical implant communication service (MICS). It allows low power transmission (output power maximum 25 μW EIRP) satisfying antenna performance compatible with the human body, assuring no interference with other radio operating in the same frequency band. For these reasons, industries are very interested to develop implanted devices operating in this band [14]. Companies such as Medtronic, Biotronic and St. Jude, have already made commercially available devices working in this band [14]. The MICS band has also been adopted by the standard IEEE 802.15.6 [15] for implanted applications. The standard IEEE 802.15.6 supports medical communication between non-invasive devices placed on the body and implanted devices placed in the body, as well as external devices that are around the body. However, this standard does not provide information on the absolute performance of the channel models. A specific task group recommended different channel models, based on seven representative scenarios [16], three of them related to implanted applications. However, in [16] it has been stated that the channel models described in the document are not intended to provide information of absolute performance and that each application can have specific requirements.

There are only few studies on implanted applications within the MICS band, since often the ISM (Industrial Scientific and Medical) band was used instead. In fact, the design of an antenna which operates in the MICS band is challenging, mainly due to limitations in the dimensions. In the MICS band the wavelength inside the human body is ~ 9 cm, while in the air is ~ 74 cm. Nevertheless, the ISM band is used for almost all the devices that we use in everyday life, such as radio-frequency identifications (RFID), Bluetooth and ZigBee, therefore the risk of interference is very high. On the contrary the MICS band has been reserved for human implantable devices, sharing the frequency only with weather balloons (400–406 MHz). In [17, 18] artificial cardiac pacemakers with implanted antennas operating in the MICS band are considered. In both studies the performance of the antennas has been analyzed by simulations with simplified phantoms. In [18] an experiment on porcine tissue is presented where it was observed that the performance was different with respect to the simulated model because of the thick fat layer and the absence of bones in the pig tissues. Additional studies have been conducted on ingested implants, such as a capsule endoscope [19, 20], and demonstrated that the MICS band is ideal for wireless implant [17]. In [20] the path loss model and the SAR level have been investigated for a spiral antenna in MICS frequencies with numerical simulations in a 3D human model. It has been demonstrated that for an endoscopy capsule the path loss exponent can vary depending on the subject (adult or child) and on the deepness in the body, i.e., on the layers of tissues between the transmitting and the receiving antenna. Therefore, it is not possible to provide a single path loss model for wireless communication for in-body to on-body and each application needs to be specifically investigated. In the case of wireless implants for recording EMG signals to drive hand prostheses, there are no studies on path loss, except our preliminary report [21].

In the present work, we propose a system based on the IEEE 802.15.6 standard [15] composed by two implanted devices that record and transmit wireless EMG signals to an on-body device positioned inside the socket of a hand prosthesis. The system can be used for each type of amputee, including subject with target muscle reinnervation (TMR), since there are no constraints for the placement of the implants. Moreover it uses a frequency band dedicated to implanted devices.

The path loss model has been investigated by FDTD (Finite Difference Time Domain) simulations, with a 3D human body model for the system proposed. Furthermore, the SAR values are investigated to demonstrate that the system is not exceeding the limitations imposed by regulations, and consequently is not harmful to humans. Finally, a preliminary evaluation of the link budget has been done taking into account the results obtained.

## Materials and Methods

**Fig 1:**
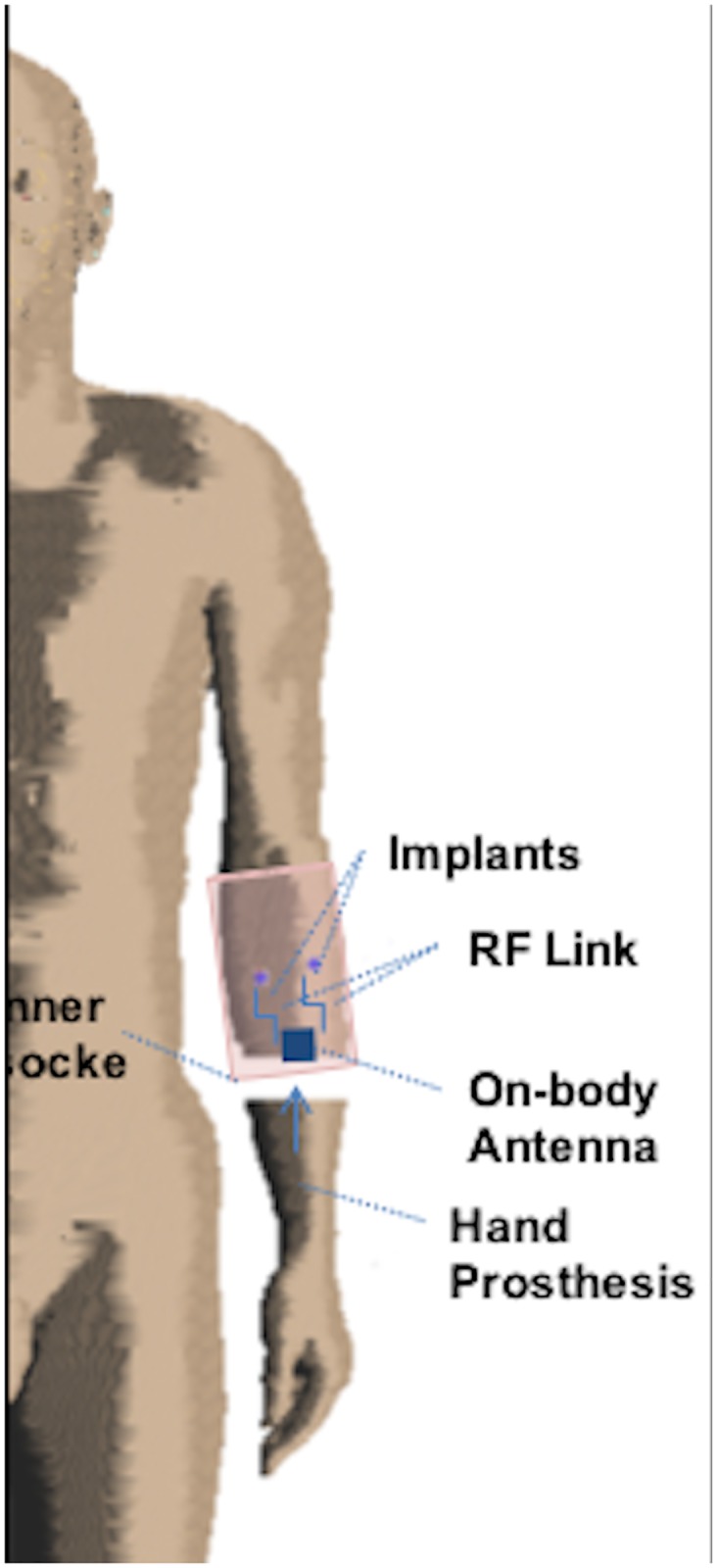
System overview.

The proposed system (Fig 1) consists of two EMG sensors implanted in the forearm of an amputee and an external device that controls the prosthesis. The implanted sensors record and process the EMG signals, which are wirelessly transmitted to an external antenna, placed on the socket prosthesis. The processed signals are then used to drive the hand prosthesis. The communication link between the devices follows the standard for Wireless Body Area Network (WBAN) [15].

### Human Model and Electromagnetic Simulation Tool

The design of wireless implant communication systems requires knowledge on the multiple layers of tissues of the human body and the respective dielectric properties. Propagation losses and SAR measurements of implanted devices cannot be investigated with *in-vivo* experiments. For this reason, numerical simulations and flat phantoms filled with muscle tissue simulating fluid are commonly used. Phantoms are non-standardized yet and they represent a simplified version of the human body [3] and not all the interfaces between the tissue layers can be represented. Moreover mimicking the limbs it is more challenging than other parts of the human body [14]. For these reasons in this study a numerical simulation has been preferred.

The EM numerical evaluations have been carried out by FDTD simulations with the 3D solver SEMCAD-X [22] and with a 3D human body model. The human body model has been obtained from magnetic resonance images (MRI) of healthy volunteers and is provided by SPEAG [23]. The model represents a man of 34 years old (denoted Duke) and it is part of the Virtual Family, which has four anatomical models (two adults and two children). These models include 80 body tissues with dielectric properties based on the database generated by Gabriel [24]. The maximum grid step of the human model is 2 mm.

### Implanted Antennas

**Fig 2:**
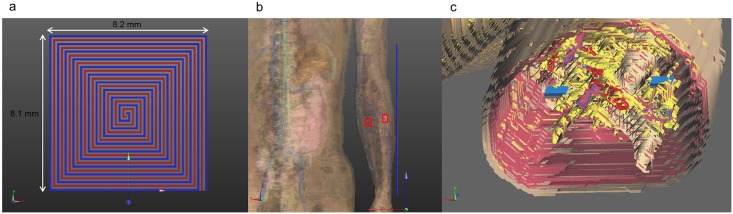
MICS implanted antenna and antenna positioning. a) front view of the implanted antenna; b) human model with the implanted antennas in the red squares and the external dipole; c) cross section of the arm with the implanted antennas.

**Fig 3:**
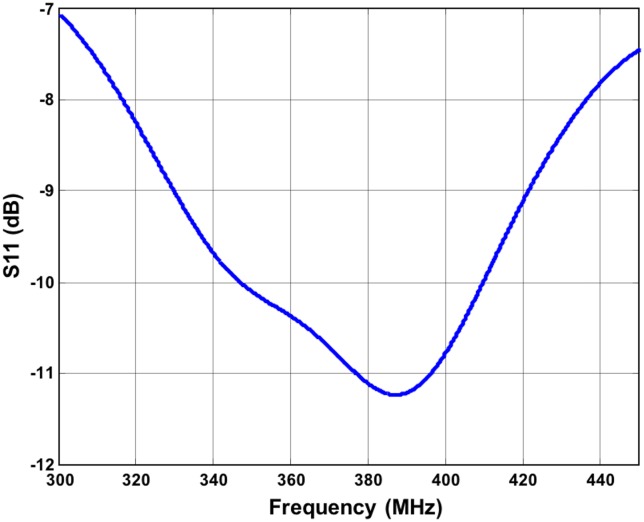
Return Loss of the implanted antenna. S_11_ is about -10 dB at 403.5 MHz.

**Fig 4:**
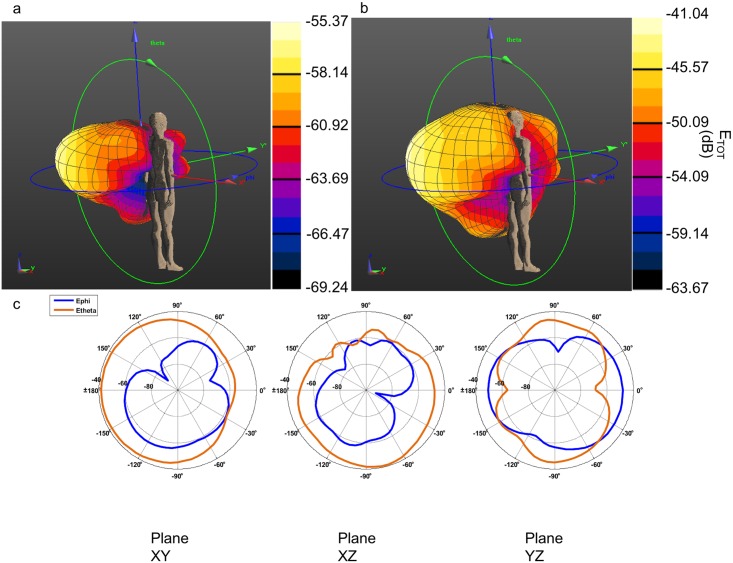
3D Gain polar and radiation pattern plots of the implanted antenna. a) 3 D gain polar plot, maximum gain equal to -55.37 dBi; b) 3D normalized radiation pattern polar plot; c) values of E_φ_ (dB) and E_θ_ (dB) in three different planes. The coordinate system is shown in figure. Maximum value -41.04 dB.

The implanted antenna selected in this work is the same used by the IEEE802.15.TG6 committee to define the channel models for the standard IEEE 802.15.6 and is extensively described in [16, 25], and [26]. The dimensions and shape of this antenna fit well in a device implanted inside a phantom limb of a transradial amputee. The antenna is composed of a single metallic layer of copper. The metallic layer is printed on a side of a D51 (NTK) substrate with dielectric constant ε_r_ = 30, loss tangent tan θ = 3.8e-05, thickness of 1 mm, and covered by RH-5 substrate with dielectric constant ε_r_ = 1.0006, loss tangent tan θ = 0 and thickness of 1 mm (Fig 2a). The antenna has been designed to operate in the human body within the MICS frequency band (402–405 MHz) [25]. Two antennas have been implanted in the left arm of the human model, approximately in the wrist extensor and flexor muscles. Fig 2b shows the implanted antennas inside the arm of Duke. A section of the arm with the implant is depicted in Fig 2c. To verify that the operating frequency in the human body is in the range of MICS band, the reflection coefficient (S_11_) of the antenna has been simulated. The return loss has been calculated after positioning the antenna inside the human body fixing the central frequency to 403.5 MHz, with the FDTD simulation platform SEMCAD X [22]. The S_11_ value was about -10 dB at 403.5 MHz as shown in Fig 3, which confirms a good impedance match. Fig 4a shows a 3D polar plot of the gain of the implanted antenna. The gain is not isotropic but varies with the direction. The maximum gain, taking into account also the losses of the body phantom, is -55.37 dBi. Fig 4b shows the simulated radiation pattern normalized to 1 V/m. The radiation pattern has been computed taking into account the impact of the human body, as recommended by [27]. The origin of the coordinate system is placed on the border of the implanted antenna. The maximum directivity is observed in the direction opposite to the x axis (in the XZ plane), but it is possible to observe (Fig 4c) that the E_θ_ and E_ϕ_ components are similar.

### On-body Antenna

**Fig 5:**
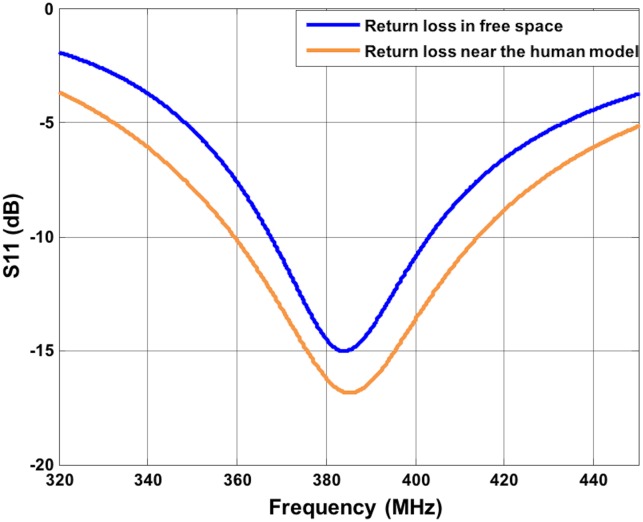
Return Loss of the dipole antenna. S_11_ is ~-12 dB at 403.5 MHz near the human body.

As a first step, to simplify the analysis and reduce the simulation time, a half-wave dipole antenna has been selected as a receiving antenna. A more realistic antenna has also been investigated as a refinement step (see below). The half-wave dipole has been modeled and tuned to operate at 403.5 MHz. Considering that the wavelength in free space at 403.5 MHz is ~74 cm, the length of each arm of the half-wave dipole has been set to 180 mm, with thickness of 2 mm and a gap between the arms of 1 mm. Fig 2b shows the location of the external antenna, close to the human body. The return loss of the dipole has been simulated in free space (without the presence of the human model) and at 1 cm from the arm surface of the human model (Fig 2b), in the near field region [28]. Fig 5 report the S11 in both conditions. The graphs are similar and show a good impedance match in the MICS band frequency (S11 at 403.5MHz is ~ -10 dB in free space and ~ -12 dB near the human model).

**Fig 6:**
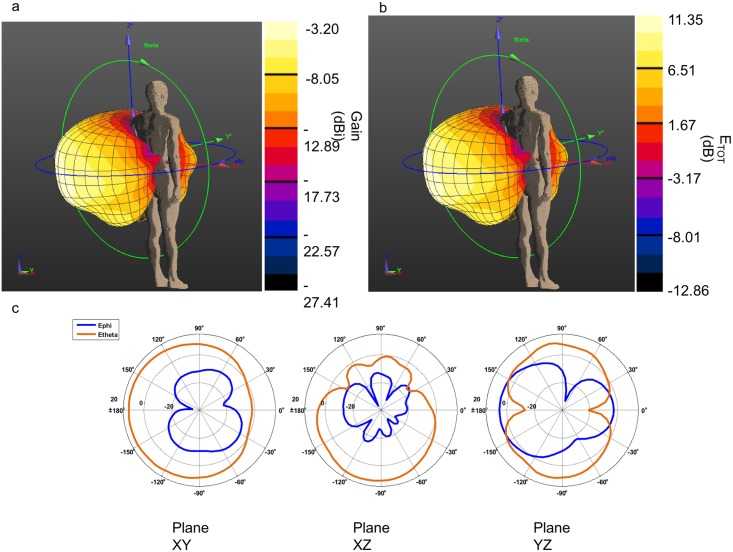
3D Gain polar and radiation pattern plots of the dipole antenna. a) 3D gain polar plot, maximum gain equal to -3.20 dBi; b) 3D normalized radiation pattern plot; c) Values of E_φ_ (dB) and E_θ_ (dB) in three different planes. The coordinate system is shown in figure. Maximum value 11.35 dB.

The gain and the radiation pattern have been computed for the external antenna in the same way and with the same coordinate system as the implanted antenna. The 3D polar plot of the gain is shown in Fig 6a and the maximum value is -3.20 dBi. The radiation pattern normalized to 1V/m is described in the Fig 6b and 6c. It has been computed taking into account the presence of the human body model. Indeed, Fig 6c shows that the E_φ_ and E_θ_ components have smaller values in the directions of the human body.

**Fig 7:**
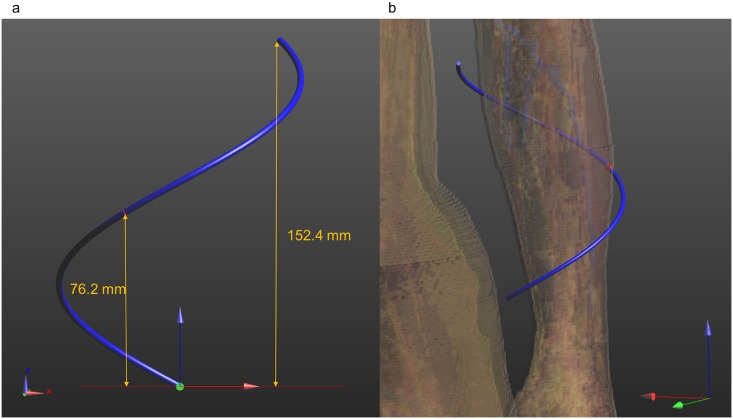
Helical antenna. a) helical antenna dimensions. b) position of the helical antenna near the human body.

**Fig 8:**
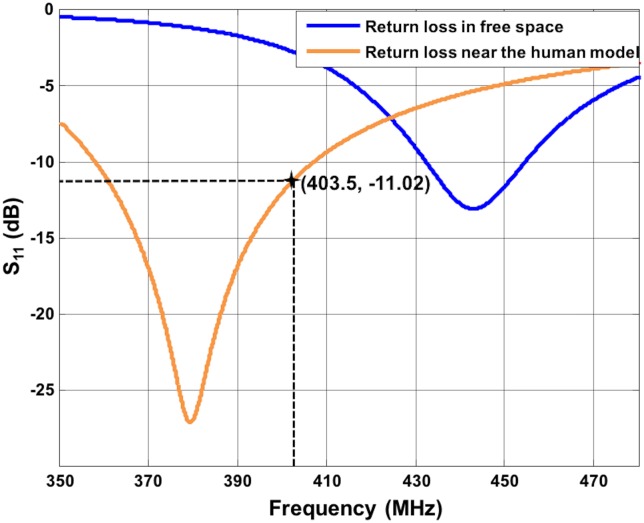
Return loss of the helical antenna. S_11_ is ~-11 dB at 403.5 MHz near the human body (orange curve).

**Fig 9:**
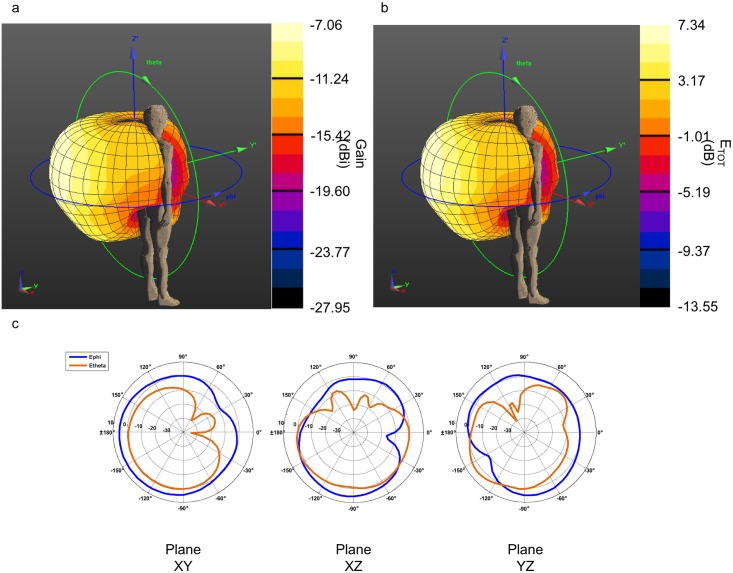
3D Gain polar and radiation pattern plots of the helical antenna. a) 3D gain polar plot, maximum gain equal to -7.06 dBi; b) 3D normalized radiation pattern polar plot; c) Values of E_φ_ (dB) and E_θ_ (dB) in three different planes. The coordinate system is shown in figure. Maximum value 7.34 dB.

A half-wave dipole as described before is very useful to analyze the electromagnetic characteristics of the communication system between the implants and the external controller of the prosthetic hand. However, considering its length, such antenna is not a good candidate to be embedded in the socket of a hand prosthesis. For this purpose, a helical dipole antenna [29] (Fig 7) with constant radius and feed at the midpoint has been designed. The helical antenna has been optimized with the FDTD simulator SEMCAD [22] to operate in the MICS frequency band when positioned near the human body. The conductor wire has a radius of 1.433 mm, the distance between the turn has been fixed to 180 mm, and the total height of the antenna is 152.4 mm (Fig 7a). The diameter of the helical is 101 mm, and the arm of the human model has approximately a diameter of 80 mm (where the implants is positioned). The antenna can be positioned around the arm as depicted in Fig 7b. The return loss (Fig 8) has been simulated in free space and near the 3D human body (as in Fig 7b). From Fig 8, it is possible to notice that there is a de-tuning of the antenna when positioned in free space. The S_11_ value at 403.5 MHz is -11 dB when the antenna is around the arm of the human body (orange curve), while S_11_ is ~-3 dB in free space at 403.5 MHz (blue curve). This shape allows the antenna to be embedded into the socket of a hand prosthesis, with gain and radiation pattern (Fig 9) similar to the half-wave dipole.

### Channel modeling for body implanted devices

The characteristics of a RF signal change during transmission between transmitting and receiving antennas. The changes depend on the environment and the distance between the transmitter and the receiver. In the case study of this work, the medium is the human body, which is a lossy heterogeneous medium with high permittivity. In-body EMG sensors controlling a prosthetic hand should rely on a good communication link, which guarantees low latency and high reliability. The first step for a good communication link is the definition of the channel modeling. A channel model allows to obtain the profile of the transmitted signal from the received signal.

Channel models are often obtained by physical measurements in experimental environments, but for practical reasons and ethical issues in the case of implanted devices this cannot be done. The simulations are in this case the means for building channel models since this allows to take into account the losses due to human body. In case of body area networks, the propagation path can be subject to fading due to shadowing by body posture, reflection, diffraction or energy absorption. The path loss represents the attenuation of the transmitted signal and in the case of body area networks it depends on the distance and frequency [16]. For implanted wireless communications, the path loss in terms of distance *d* is given by the following relation:

$$PL\left(d\right)=\frac{G_{R}P_{T}}{P_{R}\left(d\right)}$$ (1)

Where P_T_ is the transmitted power, P_R_ is the received power, and G_R_ is the gain of the receiving antenna. The transmitting antenna is considered part of the channel [3, 26, 31]. To statistically model the path loss in dB as a function of the distance, the following formula, based on the Friis formula [26, 30, 31], can be used:

$$PL_{dB}\left(d\right)=PL_{0}+10*n*{\text{log}}_{10}\frac{d}{d_{0}}$$ (2)

Where PL_0_ is the path loss in dB at a reference distance *d*_*0*_ expressed in mm, *d* [mm] is the antenna separation, and *n* is the path loss exponent, which depends on the environment where the RF signal propagates. The path loss exponent is equal to 2 in free space. Eq 2 does not take into account the shadowing component due to different body tissues and the antenna gain in different directions. Shadowing is defined as the variation of the local mean around the path loss. In particular the shadowing component takes into account the fact that there are different values of the path loss for same distance between transmitter and receiver. To take into account these losses, the path loss can be expressed as [30], [26]:

$$PL_{dB}\left(d\right)=PL_{0}+10*n*{\text{log}}_{10}\frac{d}{d_{0}}\;+S$$ (3)

where *S* is a random variable with normal distribution and standard deviation σ_s_. Indeed has been sohwn that the variation of the path loss around the average follow a log-normal distribution in many measurements [19]. *S* ~ *N*(0,σ_S_^2^) represents the shadowing component which take into account the presence of the human tissues and the antenna gain in differerent directions.

### Specific Absorption Rate (SAR)

The specific absorption rate is the rate that quantifies the RF energy absorbed in biological tissue. It is expressed in Watt per kilogram (W/kg) and it is the measure of the amount of heat generated in the antenna surrounding. Since this parameter is of extreme importance for the health of the implants carrier, there are limits and regulations that need to be fulfilled. In Europe and several countries in the world, such as Japan and Korea, the guidelines are provided by ICNIRP (International Commission on Non-Ionizing Radiation Protection) [32] which states that the local SAR averaged over a cube of 10 g of tissues should not exceed 2 W/Kg for head and trunk and 4 W/Kg for limbs. The SAR limits for the human limbs are usually lower since in arms and legs the circulatory system acts as a coolant. The guidelines provided by ICNIRP have been accepted by the International Telecommunications Union (ITU) [33] and by the World Health Organization (WHO) [34]. In US the SAR limit is 1.6 W/kg averaged over 1g of tissue [35]. No specific regulation has been issued until now for implanted devices [3], consequently we assume that the current limits for electromagnetic exposure are valid also in case of implanted wireless devices. To verify that a device respects the restrictions to electromagnetic field exposure imposed by the regulations, measurements can be done on body phantoms or mathematically modeled.

## Results and Discussions

### Channel model

The path loss has been investigated by FDTD simulations with the 3D solver SEMCAD-X [22]. The implanted antennas have been considered as the transmitters and the external antenna as the receiver. To simplify the analysis and decrease the computational time of the simulations, the half-wave dipole has been selected as the external antenna. The half-wave dipole has been placed in several positions around the arm, with a maximum distance from the skin of 10 mm and minimum distance with the implants of 25 mm (Fig 3a), to simulate possible locations for the antenna in the socket. This represents a typical scenario of transmission from in-body to body surface.

The path loss is defined in terms of the transmission coefficient (-|S_21_|_dB_) with respect to 50 Ω as the ratio of the input power at port 1 (P_in_) to the power received at port 2 (P_rec_) in a two-port setup. Considering as reference distance *d*_0_ = 25 mm and the following expression:

$$PL_{dB}\left(d\right)=-{\left|S_{21}\right|}_{dB}$$ (4)

it has been possible to model the path loss and find the values for PL_0_, *n* and *S* as defined in Eq 3. The mean value of the path loss has been obtained by fitting a least square regression line trough the scatter of measured path loss points in dB. The coefficients of the regression have been obtained with a 95% coefficient bounds.

**Fig 10:**
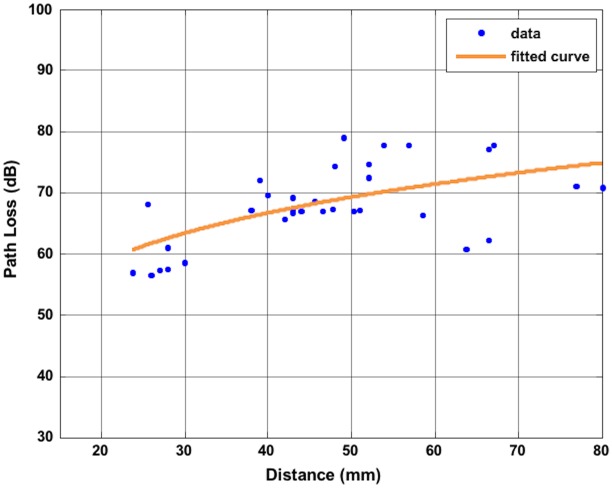
Path loss. Path loss values as function of the distance between the implanted antenna and the external antenna and representation of the fitted model.

Preliminary results have been presented by the authors in [21]. In the present work more path loss points have been added (Fig 10) respect to the results reported in [21] and the resulting parameters of the fitted simulating model are: PL_0_ = 61.12, *n* = 2.71, σ_s_ = 5.10. Fig 10 reports the values of the path loss as a function of the distance in several positions of the external antenna. The blue dots are the values of the path loss, while the orange curve is the fitting curve obtained through a least square linear regression. The model takes into account also the shadowing effect (the term S) as considered in the Eq 3. *S* is a random variable with a normal distribution, zero mean and standard deviation σ_s_ and it occurs when the distance between the two antennas is the same, but they might have different positions or directions. The distances are in the range 25–80 mm. Looking at the Fig 10, we notice that in general the path loss is increasing with the distance and the maximum value is less than 80 dB. The results obtained are in line with similar works [30], [36]. Few more simulations have been done substituting the half-wave dipole with the helical antenna. The values of the path loss obtained are in line with the model built considering the dipole. In Table 1 the values and the corresponding distances are listed.

**Table 1:** Path loss values with dipole helical antenna.

Distance (mm)	Path Loss (dB)
38	62.98
43	61.68
65	72.55
69	74.89

### Specific Absorption Rate

**Fig 11:**
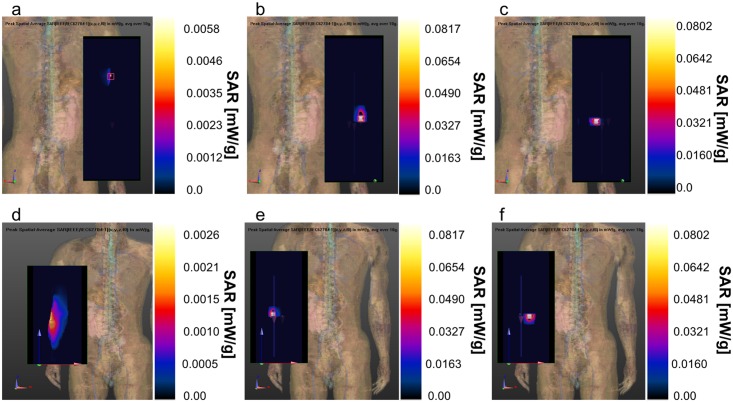
psaSAR values with the half wave dipole. a) psaSAR related to the external half-wave dipole positioned in front of the arm (scenario 1); b) psaSAR related to implant 1 (scenario 1); c) psaSAR related to implant 2 (scenario1); d) psaSAR related to the external half-wave dipole positioned on the back of the arm (scenario2); e) psaSAR related to implant 1(scenario2); f) psaSAR related to implant 2 (scenario2).

**Fig 12:**
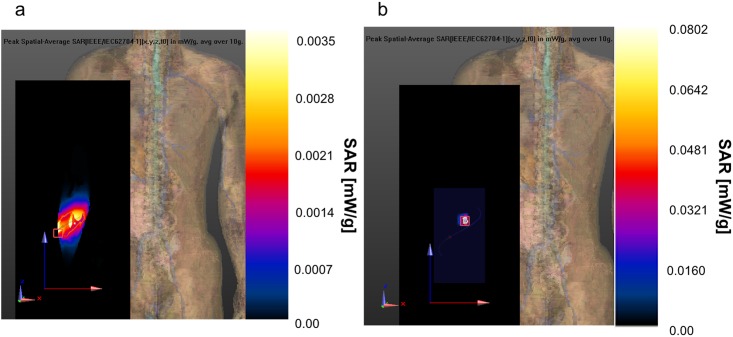
psaSAR values with the helical dipole. a) psaSAR related to the external helical dipole; b) psaSAR related to implant 1.

In this study, the induced SAR has been calculated with the software SEMCAD which uses the FDTD algorithm and a realistic human body model of an adult male (Virtual family [23]). The algorithm implemented in SEMCAD evaluates the spatial peak average SAR based on the IEEE/IEC62704-1 standard [37], which uses a cube of mass of lossy tissue (normally 1 g or 10 g) to average SAR value. We have evaluated the SAR for both the implanted antennas for two scenarios. The external antenna used for the evaluation also in this case is the half-wave dipole of Fig 3a. In a third scenario we have evaluated the SAR also with the helical antenna and one implanted antenna. In all scenarios considered it has been extracted the peak spatial average SAR (psaSAR) averaged over 10 g of tissues and normalized to 1mW input power for the implanted antennas according to [37]. In the first scenario considered the half-wave dipole is positioned in front of the arm, at ~10 mm from the skin. In Fig 11a, 11b and 11c are shown the SAR values for the external antenna and for the two implants respectively. The red square is the position of the peak value. In Fig 11a the peak has a value of 0.00577576 mW/g located on the skin of the arms where the dipole has minimum distance. For both implants the peaks (the red square) are located on the tissues that are close to the antenna. The psaSAR values are 0.0817047 mW/g (Fig 11b) and 0.0802022 mW/g (Fig 11c). All the values are lower than the limitation imposed by ICNIRP (4 W/Kg). In the second scenario the external dipole antenna is positioned in the back of the arm. The distance with the skin is also in this case around 10 mm. The psaSAR values are very similar to the previous case. The external antenna psaSAR is 0.00257913 mW/g and is located few millimeters under the skin in the back of the arm in correspondence of the dipole source (Fig 11d). The peak values of the implants are located also in this case on the tissues near them, and their psaSAR values are 0.0817043 mW/g and 0.0802018 mW/g (Fig 11e and 11f). The psaSAR values measured in the two scenarios are very close each other, infact to appreciate the differences it has been necessary to consider a precision in the order of nW/g. Moreover the values are far from the ICNRP limitations. Considering the ICNRP limitation of 4 W/kg the maximum input power allowable results ~50 mW, considering the psaSAR obtained on the implanted antennas. To complete the analysis on SAR, we have evaluated the case where the external antenna is the helical dipole presented previously. To simplify the computation and have a shorter simulation time we have considered only one implanted antenna. The values and positions of the psaSARs are depicted in Fig 12. The psaSAR for the helical dipole antenna is 0.00350877 mW/g and is located on the side of the arm, which is nearest to the source (Fig 12a). The value of the psaSAR for the implant is 0.0802028 mW/g on the tissues near the implant (Fig 12b). The values of SAR measured in this case are very similar to the other two cases and confirm that input power can be increased up to ~50 mW.

### Link budget

The feasibility of a wireless link can be validated by the link budget analysis. Given the analysis done in the previous sections a first evaluation can be provided taking into account the results obtained and the limitation provided by the ITU recommendation for MICS bandwidth [38] and by the standard for WBAN [15]. A preliminary link budget evaluation is presented in Table 2, considering a symbol rate of 151.8 kbps [15]. The influence of the human body has been taken into account in the path loss term, which includes also fading.

**Table 2:** Link budget.

PARAMETERS (up-link)	Value
Frequency	402–405 MHz
Modulation type	π/2-DBPSK
Data rate	151.8 kbps
BW Bandwidth	300 kHz
SNR	5 dB
NF Noise Figure	10 dB
L_R_ losses at the receiver	6 dB
N_0_ Thermal noise density for implant	-174 dBm/Hz
PL Path Loss (d = 45 mm) including fading	68 dB
G_T_ Transmit antenna gain	-55.37 dBi
G_R_ Receiver antenna gain (dipole)	-3.20 dBi
EIRP	-16 dBm
P_R_ Received power	-87.2 dBm
S_R_ Receiver sensitivity	-98.2 dBm
LM Link Margin	11 dB

The received power P_R_ has been calculated as:

$$P_{R}=EIRP-PL+G_{R}\;(\text{dBm})$$ (5)

where EIRP [dBm] is the effective isotropic radiated power and includes the input power and the transmitting implanted antenna gain. For MICS systems EIRP = -16 dBm [38]. PL [dB] is the path loss and it has been considered for a distance of 45 mm from the channel model obtained previously (68 dB), and G_R_ [dBi] is the receiver antenna gain. The receiver antenna considered is the half-wave dipole (G_R_ = -3.20 dBi). The resulting maximum transmit power is P_R_ = -87.2 dBm.

The receiver sensitivity is defined as:

$$S_{R}=SNR+N_{0}+BW+NF+L_{R}\;\left(\text{dBm}\right)$$ (6)

Where SNR [dB] is the signal to noise ratio, N_0_ [dB/Hz] is the thermal noise density for the implant (considering the temperature of the body 37°C), BW [dB] is the bandwidth (calculated as 10*log(BW [Hz])), NF [dB] is the noise figure, and L_R_ [dB] are the losses at the receiver. The resulting receiver sensitivity is S_R_ = -98.2 dBm.

The link margin (LM) is obtained as

$$LM=P_{R}-S_{R}\;\left(\text{dB}\right)$$ (7)

and it results LM = 11 dB.

Table 2 summarize the link budget of the system and shows that the system has a link margin of 11 dB which is very good since the gain of the transmitting antenna and the path loss can vary across subjects. Moreover the gain of the external antenna can be increased selecting a different antenna. In [38] for example it has been selected G_R_ = 2 dBi. In our case, the low gain of the implanted antenna limits the received power and the link, unless setting a quite high transmit power, which is not desirable in terms of safety (SAR limits) and battery consumption. Nevertheless the link can be improved optimizing the gain of both antennas (for example varying the orientation), especially for the external antenna, and the modulation scheme.

## Conclusions

In this study the wireless RF link between in-body EMG sensors and on-body controller for upper limb prostheses has been investigated with the limitations imposed by the standard for WBAN [15]. The channel model, including losses due to fading, has been defined in the MICS frequency band. The SAR due to the implants and to the external antenna has been computed, assuring that the values are much lower than the limitations imposed by ICNRP. Finally a preliminary link budget analysis has been performed providing a link margin of 11 dB for the implementation. These results confirm that such system can be implemented fulfilling the standard IEEE 802.15.6 for WBAN [15]. This can be interesting for industries that are developing upper limb prosthesis since they can solve some of the problems of reliability and usability that they have with the actual systems commercially available, without taking care of interference with other wireless devices. Furthermore standardized communication links can decrease the cost of the prostheses.

The study has been conducted considering only two implanted devices, but the system can be extended with as many EMG sensors as needed taking into account the results of this study. The data rate considered in the link budget analysis is 151.8 kbps with DBPSK modulation, as defined in [15]. It is possible to consider higher data rate with a different type of modulation, as also specified in [15]. The study presented here represents a first step in the development of implanted RF EMG sensors. Further analysis has to be done by considering more human body models, efficient algorithms to control the hand prosthesis able to use the information provided by the implant EMG sensors, and also a smaller antenna that can easily be fitted in the socket of the prosthesis. Nevertheless, this study has provided useful indications that can be used by researchers and manufacturers to further investigate and develop devices for controlling hand prostheses following the standard for WBAN.
